# Supplementary material for: Single-cell sequencing reveals the landscape of the human brain metastatic microenvironment
Source: Commun Biol. 2023 Jul 21;6:760. doi: 10.1038/s42003-023-05124-2 (PMC10362065; doi:10.1038/s42003-023-05124-2)
Supplement: Supplementary file 2 — Description of Additional Supplementary Files [file 42003_2023_5124_MOESM2_ESM.pdf]

Description of Additional Supplementary Files

File name: Supplementary Data 1
Description: DEGs identified for each cell cluster.

File name: Supplementary Data 2
Description: The source data behind the graphs in the paper.
