# Supplementary material for: Single-cell sequencing reveals the landscape of the human brain metastatic microenvironment
Source: Commun Biol. 2023 Jul 21;6:760. doi: 10.1038/s42003-023-05124-2 (PMC10362065; doi:10.1038/s42003-023-05124-2)
Supplement: Supplementary file 5 — Reporting Summary [file 42003_2023_5124_MOESM5_ESM.pdf]

nature portfolio

Corresponding author(s): JING SU

Last updated by author(s): Jun 18, 2023

Reporting Summary

Nature Portfolio wishes to improve the reproducibility of the work that we publish. This form provides structure for consistency and transparency in reporting. For further information on Nature Portfolio policies, see our Editorial Policies and the Editorial Policy Checklist.

Statistics

For all statistical analyses, confirm that the following items are present in the figure legend, table legend, main text, or Methods section.

n/a	Confirmed	
☐	☒	The exact sample size (*n*) for each experimental group/condition, given as a discrete number and unit of measurement
☐	☒	A statement on whether measurements were taken from distinct samples or whether the same sample was measured repeatedly
☒	☐	The statistical test(s) used AND whether they are one- or two-sided<br>*Only common tests should be described solely by name; describe more complex techniques in the Methods section.*
☒	☐	A description of all covariates tested
☒	☐	A description of any assumptions or corrections, such as tests of normality and adjustment for multiple comparisons
☐	☒	A full description of the statistical parameters including central tendency (e.g. means) or other basic estimates (e.g. regression coefficient) AND variation (e.g. standard deviation) or associated estimates of uncertainty (e.g. confidence intervals)
☐	☒	For null hypothesis testing, the test statistic (e.g. *F*, *t*, *r*) with confidence intervals, effect sizes, degrees of freedom and *P* value noted<br>*Give P values as exact values whenever suitable.*
☒	☐	For Bayesian analysis, information on the choice of priors and Markov chain Monte Carlo settings
☒	☐	For hierarchical and complex designs, identification of the appropriate level for tests and full reporting of outcomes
☒	☐	Estimates of effect sizes (e.g. Cohen's *d*, Pearson's *r*), indicating how they were calculated

Our web collection on statistics for biologists contains articles on many of the points above.

Software and code

Policy information about availability of computer code

Data collection	Our generated single-cell RNA-seq data is available in the NCBI Gene expression Omnibus database (GEO) with accession number GSE234832. External single-cell data used in this study is available in GEO with accession number GSE186344.
Data analysis	Reactome pathways and GO terms were downloaded from the MSigDB Collections. Functional enrichment was assessed by a hypergeometric test, which was used to identify a priori-defined gene set that showed statistically significant differences between two given clusters. The gene set enrichment analysis was performed by the clusterProfiler package. Test P values were further adjusted by Benjamini-Hochberg correction, and adjusted P values less than 0.05 were considered statistically significant. Differentially expressed genes (DEGs) were identified using the “FindMarkers” function in the R package of “Seurat”60. DEGs were evaluated with the Bonferroni-adjusted P-value (adj. P < 0.05) and the absolute log2-fold-change (|log2 FC| >=1). All statistical analyses are performed using R v.4.2.0.

For manuscripts utilizing custom algorithms or software that are central to the research but not yet described in published literature, software must be made available to editors and reviewers. We strongly encourage code deposition in a community repository (e.g. GitHub). See the Nature Portfolio guidelines for submitting code & software for further information.

Data

Policy information about availability of data

All manuscripts must include a data availability statement. This statement should provide the following information, where applicable:

- Accession codes, unique identifiers, or web links for publicly available datasets
- A description of any restrictions on data availability
- For clinical datasets or third party data, please ensure that the statement adheres to our policy

Our generated single-cell RNA-seq data is available in the NCBI Gene expression Omnibus database (GEO) with accession number GSE234832. External single-cell data used in this study is available in GEO with accession number GSE186344.

Research involving human participants, their data, or biological material

Policy information about studies with human participants or human data. See also policy information about sex, gender (identity/presentation), and sexual orientation and race, ethnicity and racism.

Reporting on sex and gender	The sex were reported by the participant and recorded by the physician. Patients were enrolled without considering sex and gender.
Reporting on race, ethnicity, or other socially relevant groupings	Patients were enrolled without considering race, ethnicity, or other socially relevant groupings.
Population characteristics	Five brain metastatic tissues are analyzed in this study, including three from breast primary and two from lung primary. The five patients are 41 – 63 years old (median age: 55 years), all female, and majority white with one African American patient. Two of the three breast cancer brain metastases samples have primary triple-negative breast cancers, while the other one shows ER(estrogen receptor)/PR(progesterone receptor)/HER2(human epidermal growth factor receptor 2) positive subtypes according the immunohistochemistry (for ER and PR) and in situ hybridization (for HER2). One of the lung cancer brain metastases samples has been examined without rearrangements of ALK and ROS1 and EGFR mutation. Two of the primary cancers metastasize to the left frontal lobe and three of the other primary tumors metastasize to cerebellum.
Recruitment	Patients were voluntarily enrolled in the study with written informed consent.
Ethics oversight	Acquisition of de-identified samples from the TTPSR for single-cell isolation and research use was in accordance with approved Institutional Review Board protocol.

Note that full information on the approval of the study protocol must also be provided in the manuscript.

Field-specific reporting

Please select the one below that is the best fit for your research. If you are not sure, read the appropriate sections before making your selection.

☒ Life sciences ☐ Behavioural & social sciences ☐ Ecological, evolutionary & environmental sciences

For a reference copy of the document with all sections, see nature.com/documents/nr-reporting-summary-flat.pdf

Life sciences study design

All studies must disclose on these points even when the disclosure is negative.

Sample size	Sample size has been indicated. Totally 5 patient samples are included.
Data exclusions	Single-cell RNA-sequencing data not passing the quality control was excluded.
Replication	Single cell sequencing in 10x were replicated in the two lanes. Data and findings were reproducible by re-running the analysis.
Randomization	The patients in this study is not based on randomization.
Blinding	Blinding was not relevant to this study.

Reporting for specific materials, systems and methods

We require information from authors about some types of materials, experimental systems and methods used in many studies. Here, indicate whether each material, system or method listed is relevant to your study. If you are not sure if a list item applies to your research, read the appropriate section before selecting a response.

Materials & experimental systems

n/a	Involved in the study
☒	☐ Antibodies
☒	☐ Eukaryotic cell lines
☒	☐ Palaeontology and archaeology
☒	☐ Animals and other organisms
☒	☐ Clinical data
☒	☐ Dual use research of concern
☒	☐ Plants

Methods

n/a	Involved in the study
☒	☐ ChIP-seq
☒	☐ Flow cytometry
☒	☐ MRI-based neuroimaging
